# High ω-3:ω-6 fatty acids ratio increases fatty acid binding protein 4 and extracellular secretory phospholipase A2IIa in human ectopic endometrial cells

**Published:** 2014-11

**Authors:** Korosh Khanaki, Mohammad Reza Sadeghi, Mohammad Mehdi Akhondi, Masoud Darabi, Amir Mehdizadeh, Mahdi Shabani, Ali Rahimipour, Mohammad Nouri

**Affiliations:** 1 *Reproductive Biotechnology Research Center, Avicenna Research Institute, ACECR, Tehran, Iran.*; 2 *Medical Biotechnology Research Center, Paramedical Faculty, Guilan University of Medical Sciences, Langrood, Iran.*; 3 *Monoclonal Antibody Research Center, Avicenna Research Institute, ACECR, Tehran, Iran.*; 4 *Umbilica Stem Cell Research Center, Tabriz University of Medical Sciences, Tabriz, Iran.*; 5 *Liver and Gastrointestinal Diseases Research Center, Tabriz University of Medical Sciences, Tabriz, Iran.*; 6 *Faculty of Paramedical Sciences, Shahid Beheshti University of Medical Sciences, Tehran, Iran.*; 7 *Women’s Reproductive Health Research Center, Alzahra Hospital, Tabriz University of Medical Sciences, Tabriz, Iran.*

**Keywords:** *Endometriosis*, *Secretory phospholipaseA2IIa*, *Fatty acid binding protein 4*, *n-3 polyunsaturated fatty acid*, *Cell culture*

## Abstract

**Background:**

Endometriosis, a common chronic inflammatory disorder, is defined by the atypical growth of endometrium- like tissue outside of the uterus. Secretory phospholipase A2 group IIa (sPLA2-IIa) and fatty acid binding protein4 (FABP4) play several important roles in the inflammatory diseases.

**Objective:**

Due to reported potential anti-inflammatory effects of ω-3 and ω-6 fatty acids, the purpose of the present study was to investigate the effects of ω-3 and ω-6 polyunsaturated fatty acids (PUFAs) on fatty acid binding protein 4 and extracellular secretory phospholipase A2IIa in cultured endometrial cells.

**Materials and Methods:**

Ectopic and eutopic endometrial tissues obtained from 15 women were snap frozen. After thawing and tissue digestion, primary mixed stromal and endometrial epithelial cell culture was performed for 8 days in culture mediums supplemented with normal and high ratios of ω-3 and ω-6 PUFA. sPLA2-IIa in the culture medium and FABP4 level was determined using enzyme immuno assay (EIA) technique.

**Results:**

Within ectopic endometrial cells group, the level of cellular FABP4 and extracellular sPLA2-IIa were remarkably increased under high ω-3 PUFA exposure compared with control condition (p=0.014 and p=0.04 respectively).

**Conclusion:**

ω-3 PUFAs may increase the level of cellular FABP4 and extracellular sPLA2-IIa in ectopic endometrial cells, since sPLAIIa and FABP4 may affect endometriosis via several mechanisms, more relevant studies are encouraged to know the potential effect of increased cellular FABP4 and extracellular sPLA2-IIa on endometriosis.

## Introduction

Endometriosis is an inflammatory condition defined by the presence of functional endometrial glands and stroma outside of the uterus (1). The incidence rate of this common gynecological disease is accounted for at least 10-15% of women in reproductive age (2). The common clinical symptoms are severe dysmenorrhea, pain during intercourse and pelvic pain (3). According to its severity, this disease is divided into four stages (I-IV) (4). The pathophysiology of endometriosis seems to be multifactorial and a rapidly growing body of evidences shows that probably inflammation has a critical role in the onset and development of endometriosis (5, 6).

Secretory phospholipase A2 type IIa (sPLA2IIa) is highly expressed in inflammatory conditions. sPLA2IIa is involved in the formation of eicosanoids through the hydrolysis of the sn-2 fatty acyl ester bond of membrane glycero-3-phospholipids, resulting in the release of arachidonic acid and lysophospholipids; the precursor for several pro-inflammatory molecules such as prostanoids (7, 8). The potent bactericidal property of sPLA2-IIa has been demonstrated (9). On the other hand, fatty-acid-binding proteins (FABPs) are a family of hydrophobic ligand-binding proteins such as fatty acids and eicosanoids (10, 11). Fatty acid-binding proteins (FABPs) play important roles not only in intracellular trafficking of fatty acids but also in cellular signaling, lipid metabolism, growth and differentiation, and gene transcription (12). It appears that FABP4 is involved in the inflammation through functions in both macrophages and adipocytes (13).

Eyster *et al* showed that the expression of sPLA2IIa and FABP4 were significantly up-regulated in ectopic compared with eutopic endometrium (14). Differentially expression of sPLA2IIa mRNA has been shown in peritoneal lesions of ectopic endometrium in comparison to matched eutopic endometrium from endometriosis patients (15). Several reports indicated that there is a correlation between dietary fatty acids and endometriosis pathogenesis. Coven *et al* found that in rabbits with endometriosis, given dietary fish oil containing ω-3 polyunsaturated fatty acids (PUFA_s_) attenuated the growth of endometriotic implants (16). Moreover, a prospective cohort study indicated that long-term consumption of ω-3 fatty acids was associated with decreased risk of endometriosis (17). Hence it appears that ω-3 fatty acids might be effective against inflammation in endometriosis (18).

The transcription of sPLA2 and FABP4 could be regulated by fatty acids because of existing of various functional peroxisome proliferator response elements (PPRE) in promoter region of sPLA2 and FABP4 genes (19-22). Therefore mutual association between PUFAs and sPLA2 and FABP4 may be involved in the regulation of inflammatory responses. Evaluation of the possible cross-talk between ω-3 and ω-6 PUFAs and sPLA2 and FABP4 may help to develop new approaches for controlling the disease.

Our previous study evaluated the effects of ω-3 and ω-6 fatty acids on the sPLA2IIa level in the cell lysate samples of ectopic and eutopic endometrial cells, since this type of PLA2 is secretory and has some possible biological and pathological functions in the extracellular space, we pursued to investigate and compare the effects of these fatty acids on the sPLA2IIa in the culture medium of endometrial cells (23-26).

The aim of the present study was to investigate the effects of ω-3 and ω-6 fatty acids intervention on the levels of cellular FABP4 and extracellular sPLA2-IIa in cultured ectopic and eutopic endometrial cells from patients with endometriosis. 

## Materials and methods

**Patient recruitment and specimen collection**

In this experimental study, 15 women with histologically confirmed endometriosis who had undergone diagnostic laparoscopy for pelvic pain or infertility at the Avicenna Infertility Clinic were included in this study. All patients gave informed consent and this study approved by the ethic committee of Avicenna Research Institute and performed at the institute during 2010-2011. The cases were infertile women with the age of 18-42 years (27). 

They had regular menstruation cycle that operated at secretory phase of the menstrual cycle. None of them were taking anti-inflammatory drugs during last three months before surgery. Women were excluded from the study if they had any diseases, such as endometritis, gastrointestinal or urological disease, liver or endocrine autoimmune disease, neoplastic disorders. The cases were categorized on the base of diseases stage into stage I (7 out 15) and stage II (8 out 15) according to the criteria established by American society for reproductive medicine (ASRM) (28). 

Laparoscopy and hysteroscopy techniques were performed during the same surgical intervention. Ectopic endometrial samples were removed at laparoscopy and eutopic endometrial samples were obtained through dilatation and curettage from the same patient. Ectopic tissues were removed from one of the ovaries or the peritoneum. The phase of the uterine cycle was histologically demonstrated (29). 

Ectopic and eutopic endometrial samples were immediately transferred on ice in Dulbecco's Modified Eagle Medium Nutrient Mixture F-12 (DMEM/F12) phenol red free culture media containing 100 IU/ml penicillin and 100 μg/ml streptomycin to the cell culture laboratory. Because of small sample sizes, all specimens were immediately frozen (30).

**Preparation of primary mixed stromal and endometrial epithelial cell culture**

The primary mixed stromal and endometrial epithelial cell culture was performed as previously described (23). Briefly, ectopic and eutopic endometrial tissues were simultaneously thawed and washed using serum-free culture medium. The samples of three or four patients with similar stage of disease were pooled to obtain enough number of cells for experiments; resulting in two stages I and two stage II samples of both the ectopic and eutopic endometrium. 

The tissues were gently minced into small pieces and subsequently digested using collagenase D (2 mg/ml) and DNAse I (0.05 mg/ml). Following digestion, the removal of debris and undigested tissue from cell suspension was performed through a 100µm cell strainer (31). The cell suspension was centrifuged and the cell pellet was resuspended in DMEM/F-12 and cell viability was evaluated using Trypan blue staining (32). 

Ectopic and eutopic endometrial cells were seeded at 50,000 cells/well (96-well plates, BioHit, Canada) in DMEM/F12 supplemented with NaHCO_3_ (1.2 mg/ml), penicillin (100 IU/ml), streptomycin (100 μg/ml), gentamycin (50 μg/ml), 5% fetal bovine serum (FBS) and Insulin-Transferrin-Selenite (ITS) [containing insulin (10 μg/ml ), transferrin (5.5 μg/ml) and selenite (5 ng/ml)]. 

**Figure 1:**
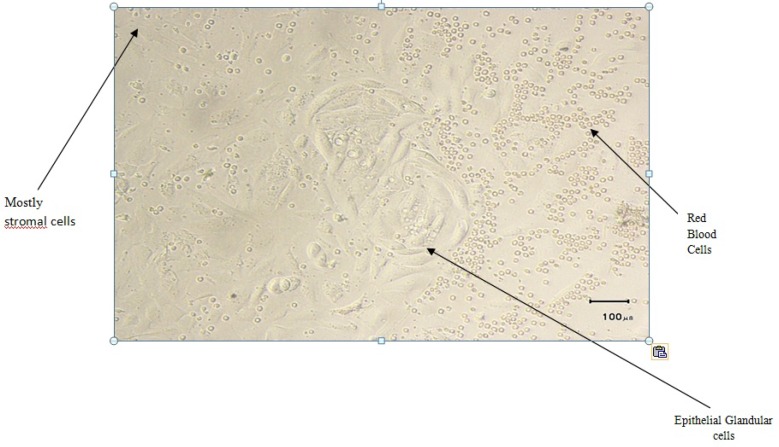
Cells resulting from endometrial tissue digestion after 3 days in primary cell culture.

**Figure 2:**
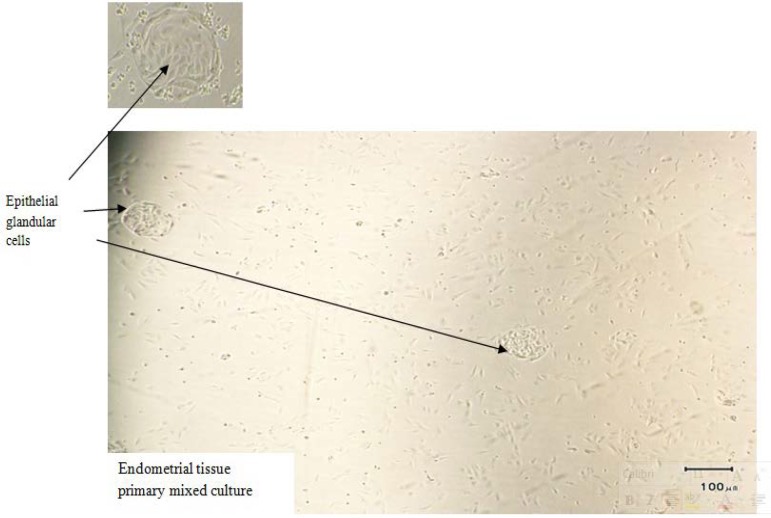
Cells resulting from endometrial tissue digestion after 5 days in primary cell culture.

The cells were incubated for 48 hr for cell attachment and after 48 hr, the culture medium was replaced with FBS-free culture medium. Then, the morphology of cultured cells was examined for presence of both tadpole-shaped glandular epithelial and fibroblast-like stromal cells using light microscopy (33). Epithelial cells were present mostly in cluster like glands, whereas stromal cells were mainly as single cells (Figure 1, 2). 

Treatment of cell culture with fatty acids was performed by removal of the culture medium after 12 hours and replaced with a fresh DMEM/F12 containing different ratios of ω-3 and ω-6 PUFAs (32). Cells were maintained in culture for 8 days more and the culture medium was refreshed after four days. The PUFAs composition was obtained from their physiological concentration in human blood (32).

**Experimental design**

Ectopic and eutopic endometrial cells were separately divided into four groups with respect to the treatment with PUFAs; (A) control group cultured in culture medium without PUFAs intervention, (B) balanced PUFAs group cultured in medium supplemented with balanced ω-3:ω-6 PUFAs ratio, (C) high ω-3:ω-6 PUFAs ratio group cultured in medium containing high amounts of ω-3 PUFAs, and (D) high ω-6:ω-3 PUFAs ratio group cultured in medium containing high amounts of ω-6 PUFAs (23). 

**The measurement of sPLA2IIa and FABP4**

The culture media was gently removed and centrifuged at the end of PUFAs treatment. The supernatants were stored at -80^o^C until measurement of sPLA2IIa. Prior to treatment and at the end of treatment with PUFAs, the cultured cells were washed using ice-cold PBS and lysed with sandwich ELISA lysis buffer [20 mM Tris-Hcl, 150 mM Nacl, 1mM disodium EDTA, 1% Triton, 25 mM sodium fluoride (NaF), 1 mM sodium vanadate (Na_3_VO_4_), and 1 mM PMSF]. The protein content of the lysates was determined with the bicinochoninic acid microplate system (BCA Protein Assay Kit, Pierce).

The level of extracellular sPLA2IIa in the conditioned media of samples and the level of cellular FABP4 in the cell lyste samples were measured using an enzyme immunoassay (EIA) procedure (Cayman Chemical kit and spibio kit respectively) and ELISA reader (Bio-Tek, Canada). The detection limit for sPLA2IIa and FABP4 was 15.6 pg/ml and 0.5 ng/ml respectively.

**Materials**

DMEM/F12, penicillin, streptomycin, DMSO, trypan blue, gentamycin, ITS, BSA, Free fatty acid BSA, Tris and fatty acids were purchased from Sigma-Aldrich(Stockholm, Sweden). Cell strainer, cell culture plates and tubes were obtained from BD Falcon Company (Heidelberg, Germany)*.* FBS was purchased from Gibco Company (Karlsruhe, Germany)*.* Collagenase D and DNAse I was obtained from Roche Company (Mannheim, Germany). 

**Statistical analysis**

The results are shown as mean±SEM. To compare the level of cellular FABP4 and extracellular sPLA2-IIa between and within ectopic and eutopic groups, statistical analysis was performed by the Kruskal-Wallis and Mann-Whitney tests. To assess correlation between FABP4 and sPLA2IIa, correlation bivariate test was performed. Significance difference was accepted at p<0.05.

## Results

The level of sPLA2IIa in the culture medium and FABP4 and level of total cell proteins showed the large specimen to specimen variation; therefore, the results of sPLA2IIa and FABP4 were normalized according to total protein of cell lysate and expressed as percentages of sPLA2IIa and FABP4 level under control condition on day eight after intervention.

**The results of FABP4**

**Figure 3:**
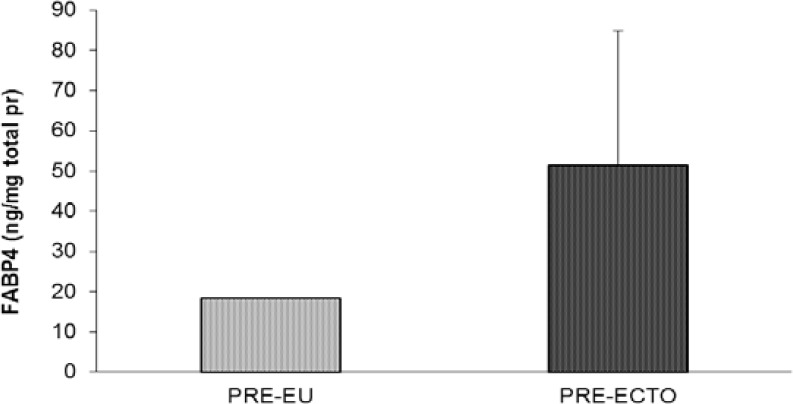
The FABP4 level in the two cells groups (ectopic and eutopic endometrial cells) from the endometriosis patients under pretreatment condition.

ECTO: Ectopic; EU: Eutopic; PRE: pretreatment condition; FABP_4_: Fatty acid binding protein 4 (ng/mg total protein); Data are shown as Mean± SEM.

Under pre-treatment condition, the level of FABP4 in the ectopic endometrial cells group was higher than the eutopic group although not statistical significant (51.303±33.506 vs. 18.236±0.05141 ng/mg total protein) (Figure 3). Comparing to control PUFAs group, the FABP4 level was different (although no statistically significant) between ectopic and eutopic endometrial cells for each of the three matched treatments (balanced, high ω-3 and high ω-6 PUFAs groups.

**Figure 4:**
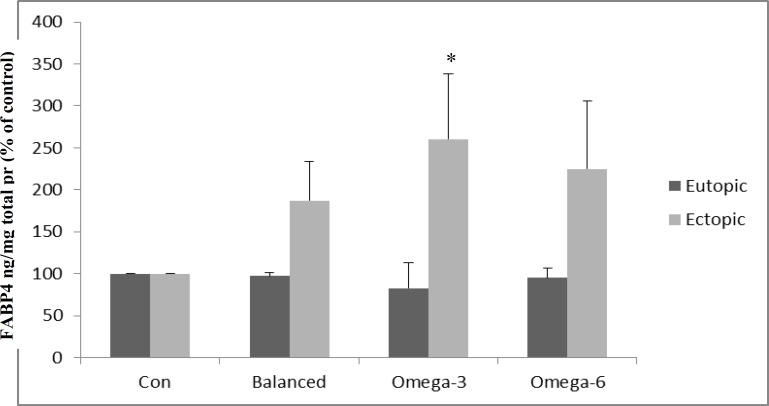
The FABP4 level in the two cells groups (ectopic and eutopic endometrial cells) from the endometriosis patients under matched culture treatments.

Within ectopic group, FABP4 level remarkably increased during high ω-3 intervention compared with control condition (260.35%±77.33% of control value; p=0.014); The FABP4 level was higher in balanced and high ω-6: ω-3 PUFAs groups comparing to control PUFAs group (187.04%±46.49 % and 224.99%±80.92% of control value, respectively) (Figure 4). The level of FABP4 was similar in control and each of the three PUFAs groups in eutopic endometrial cells (Figure 4).

**The results of sPLA2IIa**

The level of sPLA2IIa in the culture medium was similar in control and balanced PUFAs groups in both ectopic and eutopic endometrial cells. Comparing to balanced PUFAs group, the secreted sPLA2IIa level was different between ectopic and eutopic endometrial cells in both high ω-3:ω-6 PUFAs and high ω-6:ω-3 PUFAs groups. Although the level of sPLA2IIa in the culture medium was higher in eutopic endometrial cells comparing to ectopic cells in high ω-6:ω-3 PUFAs group, this difference was not statistically significant. 

**Figure 5:**
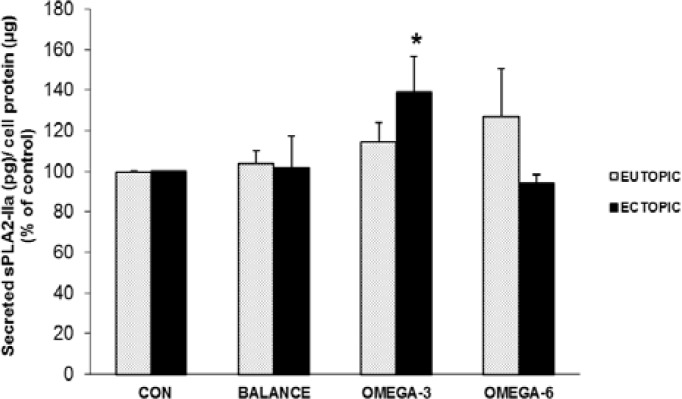
The cell secreted sPLA2IIa level in two ectopic ‎and eutopic endometrial cells from patients with endometriosis under ‎matched culture interventions.

Interestingly, within ectopic group, the sPLA2IIa level in the culture medium significantly increased under high ω-3 intervention compared with control condition (139.3%±17.411 % of control value, p<0.05). Another finding was that both high ω-3:ω-6 and ω-6:ω-3 PUFAs ratio lead to increasing in secreted sPLAIIa of eutopic endometrial cells in comparison to control and balanced ω-3:ω-6 PUFAs conditions (114.56%±9.4% and 127.28%±23.4% of control value, respectively). Finally, there was no significant difference in the level of sPLA2IIa in the culture medium between ectopic and eutopic group under each of the three matched PUFAs treatments (Figure 5). FABP4 level showed a direct correlation (although no statistically significant) with sPLA2IIa level (r=0.29; p=0.17).

FABP_4_: Fatty acid binding protein 4 (ng/mg total protein, % of control); Con: control, Balanced: balanced ω3: ω6, Omega-3: High ω3: ω6, Omega-6: High ω6: ω3. Data has been shown as Mean±SEM. * p<0.05 compared with control within group.

sPLA2IIa: secretory phospholipase ‎A2IIa (pg/µg cell protein, % of control), CON: control, BALANCE: ‎balanced ω3:ω6, OMEGA-3: High ω3: ω6, OMEGA-6: High ω6: ω3. ‎Data is presented as Mean± SEM. *: P<0.05 compared with control within group.‎

## Discussion

Up-regulation of sPLA2IIa and FABP4 gene expression in ectopic versus matched eutopic endometrium from endometriosis patients was reported in previous studies (14, 15, 34). The present study was designed to assess the cellular FABP4 and extracellular sPLA2-IIa in cultured ectopic and eutopic endometrial cells from endometriosis cases under treatment with balanced and high ratios of ω-3 and ω-6 PUFAs. By obtaining ectopic and eutopic endometrial specimens from the same patient, the influence of potential confounding factors such as the day of menstrual cycle, nutrition and genetic background was eliminated in our study. In addition, all samples were obtained in the secretary phase, leading to the minimal hormonal changes. For maintaining paracrine regulation and cell interaction, primary mixed stromal and epithelial cell culture seems to be better model (35).

FABP4 level was higher (although not statistically significant) in ectopic compared to eutopic group prior to PUFAs treatments, in view of the fact that FABP4 is largely expressed in adipocytes and macrophages, this result may be due to the macrophages with main ectopic endometrial cells (13). Eyster *et al *study demonstrated that FABP4 expression was considerably increased in ectopic versus matched eutopic endoetrium from endometriosis patients (14). Zaerakas *et al* also found that the expression of FABP4 was dramatically increased in endometriotic tissue compared with normal endometrium from endometriosis patients (34). At the above mentioned studies, the endometriosis cases were in the stages of moderate and severe while in the current study, the minimal and mild stages of endometriosis were diagnosed.

Within ectopic group, FABP4 level was significantly increased in the presence of high ω-3: ω-6 PUFAs ratio compared with control medium. In line with this finding, our previous study showed that the level of secretory phospholipase A2IIa (sPLA2IIa) in ectopic endometrial cells was considerably increased particularly under high ω-3 ratio exposure compared to control medium (23). In addition, regardless of different sampling design, this result was in part, similar to the *in vitro *study conducted by Gazvani *et al *in which the level of IL-8 (a proinflammatory cytokine) in endometrial cell culture from women with endometriosis was remarkably increased specially by high ω-3:ω-6 PUFAs ratio compared with control condition (32). 

FABP4 acts as transport vehicle for moving lipid molecules across cellular membranes, in this manner; it enhances the access of secretory PLA2 IIa to its substrate (36). Therefore the present result might be related in part to the sPLA2IIa result (23). It is likely that the increased FABP4 level in ectopic cells and therefore increased available substrate for sPLA2IIa, may increase sPLA2IIa affinity to the ectopic endometrial cells. It has been suggested that FABP4 by binding and targeted transferring long chain fatty acids such as ω-3 fatty acids to their receptor (PPAR-gamma) in the nucleus, directly increases the PPAR-gamma activity, thereby allowing ω-3 PUFAs to exert their gene transcriptional regulation (37).

It has been reported that loss of FABP4 is related to carcinoma progression; hence, FABP4 is suggested as a tumor suppressor protein (38, 39). Although endometriosis is not considered to be malignant, but locally is invasive (14). Therefore with respect to the growth inhibitory action of FABP4, it is likely the production of FABP4 in endometriosis has an antiproliferative effect (40). In addition, FABP4 has a role in causing apoptosis through inducing up-regulation of pro-apoptotic factors or down-regulation of vital growth factors (41).

High ω-3 exposure significantly increased the level of sPLA2IIa in the culture medium in ectopic endometrial cells from women with endometriosis. This finding was verified by our previous study on treated cell lysate samples where especially high ω-3: ω-6 ratio significantly increased the cell associated sPLA2IIa level in the ectopic endometrial cells compared to control condition (23). It seems that the stimulation of sPLA2IIa production by exposure to different ratio of ω-3 and ω-6 PUFAs is better reflected in cell associated compartment compared with the cell secreted compartment. Our findings in eutopic cells were similar to Gazvani *et al* study that found eutopic endometrial cells from women without endometriosis secreted higher concentrations of IL-8 in the presence of high ω-3 and high ω-6 compared to control condition (32).

The increased level of sPLA2 can lead to the increased release of arachidonic acid and eicosanoides synthesis and eventually increased inflammatory reactions; this effect is in contrast to the useful effect of ω-3 PUFAs on endometriosis (7, 8). 

It is well documented that sPLA2 IIa binds and degrades preferably outer plasma membrane containing high level of anionic phospholipids due to having the positive charged binding surface (9, 42-44). Inflammation impacts the cellular membrane situation and leads to elicit negatively charged membrane phospholipids (45). Perhaps sPLAIIa with effect on negatively charged phospholipids induces the hydrolytic degradation of inflammatory membrane phospholipids. If this mechanism is proven, this effect could be considered in consistency with beneficial effects of ω-3 PUFAs on endometriosis.

It has been demonstrated that oxidative cell injury plays a key role in initiation and progression of endometriotic damage (46, 47). The role of sPLA2 in the detoxification of oxidized membrane phospholipids has been demonstrated, alternatively sPLA2 acts as membrane phospholipid repair enzyme; as the first step in the repair process for oxidative damage to membrane lipids, this enzyme preferably hydrolyses oxidized fatty acids (48-50). This function of sPLA2 is in line with beneficial effects of ω-3 PUFAs on endometriosis. It is likely that sPLA2IIa by reducing oxidative stress and repairing the membrane phospholipids following endometriotic damage could decrease the proliferation of the damaged cells, although we didn’t evaluate this complicated process and needs to be investigated.

It has been proposed that sPLA2IIa plays an essential physiological function in the scavenging and clearance of pathological cell debris containing microparticles generated due to the inflammatory reaction (51). Thus an anti-inflammatory (resolving) role of sPLA2IIa in the inflammatory reaction has been considered (51, 52). 

Overall, it appears that sPLAIIa and FABP4 could affect endometriosis via different mechanisms. Some of the effects seem to be in agreement with the beneficial effects of ω-3 PUFAs on endometriosis and some of the effects might be in opposition to the beneficial effects of ω-3 PUFAs on endometriosis (17, 18). Further investigations are required in this area.

## Conclusion

ω-3 PUFAs may increase the level of cellular FABP4 and extracellular sPLA2-IIa in ectopic endometrial cells, since sPLAIIa and FABP4 may affect endometriosis via several mechanisms. More relevant studies are encouraged to know the potential effect of increased cellular FABP4 and extracellular sPLA2-IIa on endometriosis.
